# Development and preliminary validation of a leadership competency instrument for existing and emerging allied health professional leaders

**DOI:** 10.1186/s12913-016-1301-1

**Published:** 2016-02-19

**Authors:** Hui-Gek Ang, Jeremy Meng-Yeow Koh, Jeffrey Lee, Yong-Hao Pua

**Affiliations:** Allied Health Division, Singapore General Hospital, Singapore, Singapore; Department of Physiotherapy, Singapore General Hospital, Singapore, Singapore

## Abstract

**Background:**

No instruments, to our knowledge, exist to assess leadership competency in existing and emerging allied health professional (AHP) leaders. This paper describes the development and preliminary exploration of the psychometric properties of a leadership competency instrument for existing and emerging AHP leaders and examines (i) its factor structure, (ii) its convergent validity with the Leadership Practices Inventory (LPI), and (iii) its discriminative validity in AHPs with different grades.

**Methods:**

During development, we included 25 items in the AHEAD (Aspiring leaders in Healthcare-Empowering individuals, Achieving excellence, Developing talents) instrument. A cross-sectional study was then conducted in 106 high-potential AHPs from Singapore General Hospital (34 men and 72 women) of different professional grades (49 principal-grade AHPs, 41 senior-grade AHPs, and 16 junior-grade AHPs) who completed both AHEAD and LPI instruments. Exploratory factor analysis was used to test the theoretical structure of AHEAD. Spearman correlation analysis was performed to evaluate the convergent validity of AHEAD with LPI. Using proportional odds regression models, we evaluated the association of grades of AHPs with AHEAD and LPI. To assess discriminative validity, the c-statistics – a measure of discrimination – were derived from these ordinal models.

**Results:**

As theorized, factor analysis suggested a two-factor solution, where “skills” and “values” formed separate factors. Internal consistency of AHEAD was excellent (α-values > 0.88). Total and component AHEAD and LPI scores correlated moderately (Spearman ρ-values, 0.37 to 0.58). The *c*-index for discriminating between AHP grades was higher for AHEAD than for the LPI (0.76 vs. 0.65).

**Conclusion:**

The factorial structure of AHEAD was generally supported in our study. AHEAD showed convergent validity with the LPI and outperformed the LPI in terms of discriminative validity. These results provide initial evidence for the use of AHEAD to assess leadership competency in AHPs.

**Electronic supplementary material:**

The online version of this article (doi:10.1186/s12913-016-1301-1) contains supplementary material, which is available to authorized users.

## Background

Leadership plays a central and critical component in managing patients toward optimal clinical outcomes, and in recent years, there has been an increasing emphasis on the assessment of leadership performance. In turn, the accurate assessment of leadership performance plays a vital role in the development and improvement of leadership in healthcare organizations.

Leadership competencies are defined as leadership skills and behaviors that contribute to superior performance. By using a competency-based approach to leadership, organizations can better identify and develop their next generation of leaders [1]. Reviewing the literature, most studies on leadership [2] have focused on supervisory leadership of *existing* leaders and their immediate followers [3]. Few studies, however, have examined the leadership assessment of *potential* leaders, and to our knowledge, no studies have done so on healthcare professionals.

Because there is no validated and reliable instrument to holistically assess leadership competency in emerging and existing leaders in healthcare, we developed a leadership competency instrument which we have called the AHEAD (Aspiring leaders in Healthcare - Empowering individuals, Achieving excellence, Developing talents) instrument.

## Methods

### Study design and participants

A cross-sectional survey design was used in this study. Data was collected from July 2013 until February 2014 at the Allied Health Division of the Singapore General Hospital. In total, 106 AHPs (34 men and 72 women) of different professional grades (49 principal-grade AHPs, 41 senior-grade AHPs, and 16 junior-grade AHPs) completed both the AHEAD and LPI instruments. These 106 AHPs are come from Dietetics, Medical Social Services, Occupational Therapy, Physiotherapy, Pharmacy, Podiatry and Speech Therapy departments. Department heads selected promising AHPs with the potential for growth and development, as well as existing leaders in their respective department. This study was exempted from review by the SingHealth Centralised Institutional Review Board.

### Instrument development

To ensure that AHEAD items are applicable to all within the division, we first conducted item-generation steps through a literature review. This was followed by item-reduction using pretesting and input from discussion groups. The Leadership Practices Inventory [4] served as an intellectual and structural model during the development of AHEAD.

#### Item generation and reduction

To identify and generate items appropriate for a leadership competency instrument, we used 2 strategies: We conducted a systematic review of literature [5–8], and obtained input from relevant heads of departments on the competency domains, items and descriptors related to the knowledge, skills and values associated with effective leadership. Shortlisted items and descriptors were then categorized under 2 domains – Values and Skills. All items were rated using a 5-point Likert scale where 1 = not ready, 2 = somewhat ready, 3 = usually ready, 4 = often ready, and 5 = always ready. The instrument was then pretested with a group of 10 AHPs from 4 departments to examine item relevance and question clarity. Based on pretest findings, the total number of items was reduced from 43 to the final 25 items (Additional file 1).

### Instrument validation

The aim of this phase was to examine the factorial, convergent, and discriminative validity of the AHEAD instrument. To this end, each participant completed both the AHEAD and the LPI instruments through self-evaluation, followed by a facilitated discussion with their supervisor to reach an agreed score. In addition, participants were provided a resource guide which contained descriptors and suggested developmental resources. The subsequent scores were then sent to the team for collation.(Additional file 2)

In our study, factorial validity was examined by assessing the extent to which the items under “skills” and “values” conformed to their respective theorized constructs [9]. Cross-sectional convergent validity was examined by assessing the extent to which AHEAD was associated with the LPI (described later). Discriminative validity – the ability of an instrument to discriminate between known groups, was examined by assessing the extent to which AHEAD discriminated AHPs of different professional grades – namely, in ascending order, junior, senior, principal, and senior principal grades.

#### Leadership practices inventory

Cross-sectional convergent validity was examined by assessing the extent to which AHEAD was associated with Kouzes and Posner’s Leadership Practices Inventory (LPI) [10]. The LPI consists of two instruments; the LPI-Observer for the subordinates of the individual that is being assessed to report on their supervisor, and the LPI-Self for the individual or supervisor to self-report. Due to its similarity in assessment format with the AHEAD instrument, only the LPI-Self instrument was used. LPI-Self contains 30 questions covering 5 dimensions of the LPI (Challenging, Inspiring, Enabling, Modeling, and Encouraging). Responses are made on a 5-point Likert-type scale, and a total score ranging from 30 to 150. A higher score would indicate stronger self-perceived leadership skills. Internal reliability coefficients for the LPI-Self ranged between .75 and .87 (mean α = .81) [10, 11].

### Statistical analysis

We used descriptive statistics to characterize the study sample: we used means with SDs and medians with IQRs for continuous variables and frequencies with percentages for categorical variables. To examine the factorial validity of AHEAD, we used exploratory factor analysis (EFA). Given that no prior analyses have been performed on AHEAD, EFA is an appropriate first-step technique to explore the nature and number of factors that account for the covariation between the items [12]. We performed EFA on the polychoric correlation matrix of the ordinal-scaled AHEAD items, and we used ordinary least squares extraction with oblique (oblimin) rotation. Of note, an oblique rotation was used because we expected “Values” and “Skills” to be correlated. To determine the number of factors to be extracted, we relied on 3 principles: (i) inspection of the scree plot, (ii) interpretability of the factors, and (iii) results from Horn’s parallel analysis which uses the “mean eigenvalue criterion” [13–15]. Finally, we examined the internal consistency of each factor using Cronbach’s alpha [9, 16].

To examine convergent validity, we computed Spearman correlation between AHEAD and LPI. To examine the discriminative validity of AHEAD and LPI in differentiating AHPs of different professional grades – an ordinal outcome with 4 categories – we computed the rank-biserial correlations which described the correlations of AHP grades with AHEAD and LPI. To statistically compare discriminative validity of AHEAD and LPI, we used separate proportional odds models and regressed AHP grades on AHEAD and LPI [17]. From each ordinal model, we computed the concordance index (*c*-index), where a value of 1 represents perfect discrimination and 0.5 represents chance discrimination. Next, we used a bootstrapping procedure (1000 repetitions) to calculate the difference in *c*-indices between AHEAD and LPI models. Specifically, by subtracting the *c*-index of the LPI from that of AHEAD, positive values indicated that the discriminative validity index of AHEAD was superior to those of LPI.

All statistical analyses were done with *R* software, version 3.0.1, using the *psych* [18] and *rms* [19] packages.

## Results

Table 1 shows the characteristics of participants who were involved in the AHEAD instrument validation phase. The participants were predominantly female (68 %) and had, on average, 12 years of working experience. Amongst the 106 participants, 16 (15 %) were junior AHPs, 41 (39 %) were senior AHPs; 37 (36 %) were principal AHP; and 12 (12 %) were senior principal AHPs.

**Table 1:** Characteristics of AHEAD instrument participants

*N* = 106	
Gender, N (%)	
Female	72 (67.9)
Male	34 (32.1)
Mean age (SD), years	34.1 (0.545)
Median working experience since graduation (IQR), years	10 (7)
Healthcare profession, N (%)	
Dietetics	6 (5.7)
Medical social services	18 (17.0)
Occupational therapy	4 (3.8)
Pharmacy	20 (18.9)
Physiotherapy	43 (40.6)
Podiatry	8 (7.5)
Speech therapy	7 (6.6)
Median number of Staff Members Supervised (IQR)	5 (7.5)

Table 2 shows the results of the EFA. EFA and parallel analyses suggested a two-factor solution which explained 49 % of the total variance. Consistent with the original instrument and theory, we named the two factors “Values” and “Skills.” Overall, all AHEAD items had a minimum factor loading of 0.40 and 4 items showed cross loadings (that is, factor loadings exceeded 0.30 on both factors). Contrary to our original hypothesized model, 4 items did not load on their designated factors – namely, items Value 4 (“Courage”), Value 7 (“Inspiration”), Value 9 (“Perseverance”), and Skill 11 (“Interpersonal Skills”). Cronbach alpha values for “Values” and “Skills” factors were 0.88 (95 % CI, 0.88 to 0.95) and 0.93 (95%CI, 0.90 to 0.97), respectively, indicating excellent internal consistency. Cronbach alpha value for the whole AHEAD instrument was 0.95 (95 % CI, 0.92 to 0.97), indicating that it is appropriate to compute a total score for AHEAD.

**Table 2:** Descriptive statistics and exploratory factor analysis

			Factor analysis	
		Mean (SD)	Factor 1 (Skills)	Factor 2 (Values)
Values				
Value 1	Collegiality	3.63 (0.76)	0.11	0.90
Value 2	Commitment	3.67 (0.75)	0.36	0.42
Value 3	Compassion	3.71 (0.73)	−0.08	0.96
Value 4	Courage	3.17 (0.83)	0.66	0.10
Value 5	Humility	3.63 (0.71)	0.29	0.45
Value 6	Impartiality	3.62 (0.79)	0.36	0.45
Value 7	Inspiration	2.81 (0.91)	0.71	0.18
Value 8	Integrity	3.88 (0.80)	0.20	0.52
Value 9	Perseverance	3.43 (0.83)	0.42	0.23
Skills				
Skills 1	Change Mgt	2.98 (0.93)	0.64	0.02
Skills 2	Creativity	2.93 (0.92)	0.57	0.14
Skills 3	Decision Mkg	3.18 (0.86)	0.60	0.09
Skills 4	Budget Mgt	2.28 (0.97)	0.66	−0.22
Skills 5	Org Awareness	2.84 (0.90)	0.77	−0.06
Skills 6	Talent Mgt	2.96 (1.01)	0.81	−0.001
Skills 7	Project Mgt	3.08 (0.85)	0.74	−0.04
Skills 8	Facilitation	3.08 (0.93)	0.83	0.09
Skills 9	Mentoring	3.39 (0.99)	0.61	0.24
Skills 10	Motivation	3.10 (1.00)	0.78	0.12
Skills 11	IPerson Skills	3.29 (0.82)	0.36	0.53
Skills 12	Inquiry	3.20 (0.91)	0.50	0.36
Skills 13	Presentation	3.14 (0.87)	0.55	0.19
Skills 14	Writing	2.98 (0.92)	0.63	−0.06
Skills 15	Foresight	2.55 (0.94)	0.89	−0.28
Skills 16	Knowledge	3.35 (0.93)	0.70	0.10

**Fig. 1:**
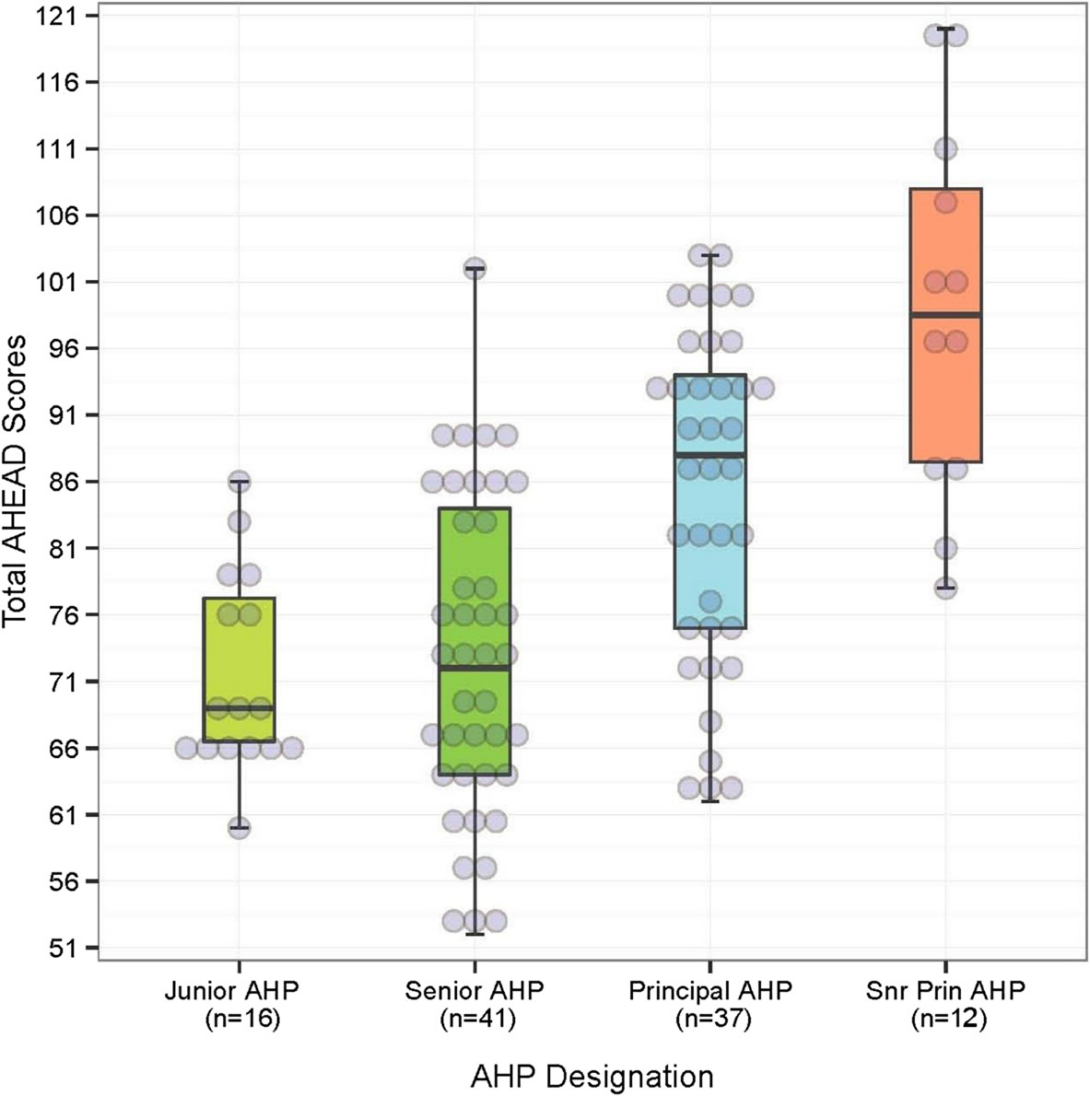
Boxplots of AHEAD scores by AHP Designation. Box-and-whisker plots of total AHEAD scores by designation of Allied Health Professionals (AHPs). In each box and whisker plot, the lower and upper edges of the box indicate the 25th and 75th percentiles; the horizontal black line indicates median; the endpoints of the notch define the CI of the median; and the whiskers indicate the highest and lowest values that are within 1.5 times the interquartile range from the box hinge. The circles represent the AHEAD scores of individual AHPs (a jitter effect has been incorporated to improve visualization of points)

**Fig. 2:**
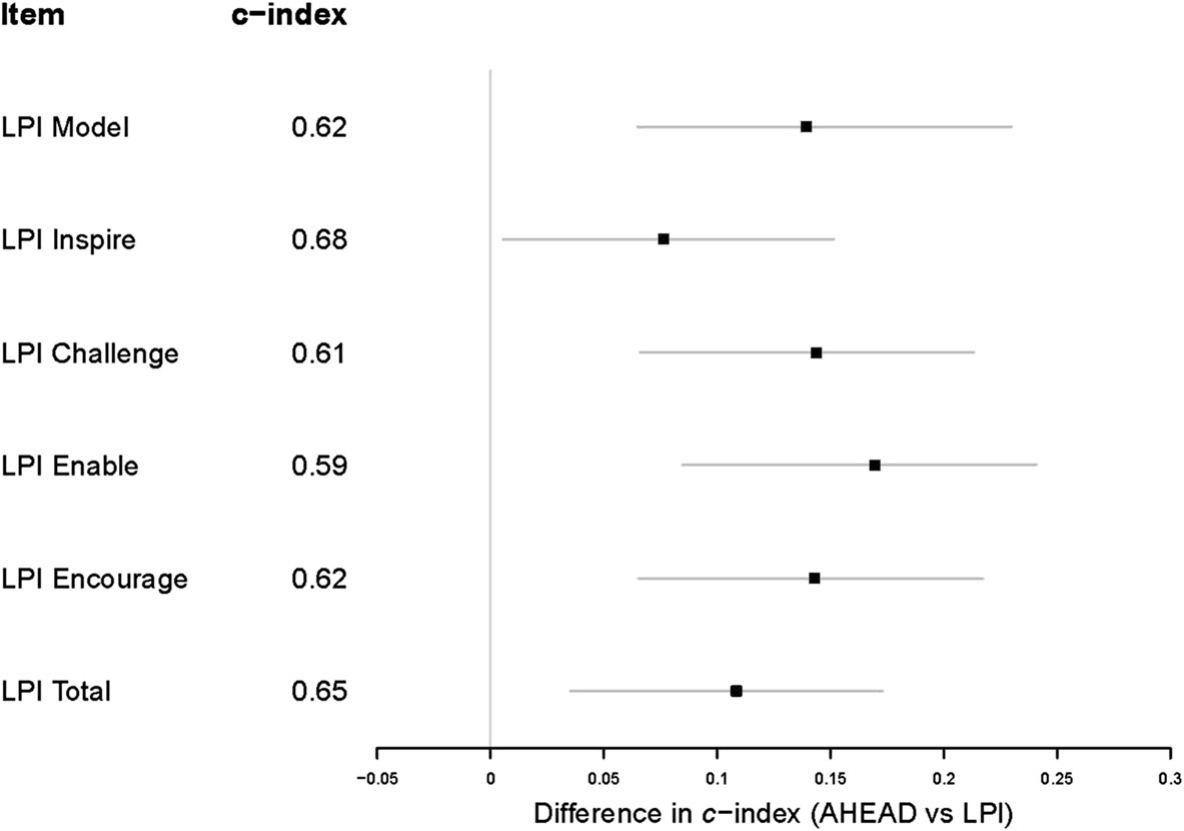
Discriminative Validity. Comparison in discriminative validity between the AHEAD (0.76) and LPI (component and total scores) instruments. Discriminative validity is indexed by the c-statistic -a measure of discrimination. Bootstrap 95 % CIs for the differences in c-statistic were bias-corrected and derived using 1000 replications

Table 3 shows the correlations among the component and total measures of AHEAD and LPI. Convergent correlation coefficients between the two instruments were on the order of 0.37 to 0.57, and were statistically significant. As regards discriminative validity, with the exception of the LPI-Enable scores, rank-biserial correlations revealed weak associations between AHP grades and LPI scores (rho values, 0.19 to 0.31). In contrast, rank-biserial correlations between AHP grades and AHEAD scores were stronger and moderate in magnitude (rho values, 0.47 to 0.55, *P*-values < 0.001). Fig. 1 shows the distribution of AHEAD scores across the different AHP grades. Proportional odds regression further supported the discriminative validity of the AHEAD instrument. Specifically, the *c*-index for discriminating AHP grades was higher for the total AHEAD scores than for the total LPI scores (0.76 vs. 0.65; bootstrap 95%CI for difference, 0.04 to 0.18). Fig. 2 summarizes the differences (and bootstrap 95%CI) in *c*-indices between the total AHEAD scores and the LPI (component and total) scores.

**Table 3:** Spearman correlation matrix

	(1)	(2)	(3)	(4)	(5)	(6)	(7)	(8)	(9)
Seniority (1)									
AHEAD Values (2)	0.50***								
AHEAD Skills (3)	0.55***	0.76***							
AHEAD Total (4)	0.56***	0.87***	0.98***						
LPI Model (5)	0.26**	0.52***	0.52***	0.55***					
LPI Inspire (6)	0.39***	0.40***	0.54***	0.52***	0.65***				
LPI Challenge (7)	0.25**	0.39***	0.53***	0.50***	0.61***	0.70***			
LPI Enable (8)	0.19*	0.45***	0.37***	0.41***	0.58***	0.46***	0.58***		
LPI Encourage (9)	0.26**	0.48***	0.44***	0.48***	0.70***	0.64***	0.51***	0.61***	
LPI Total	0.33***	0.51***	0.57***	0.58***	0.83***	0.86***	0.82***	0.73***	0.83***

*P < 0.05 **P < 0.01 ***P < 0.001

## Discussion

In a sample of 106 emerging and existing AHP leaders from 7 professions, the factorial structure of the AHEAD instrument was generally supported. AHEAD showed convergent validity with the LPI. It also showed better validity for discriminating AHP grades than did the LPI. To our knowledge, AHEAD is the first validated specific instrument to measure leadership competency in existing and emerging AHP leaders.

Consistent with our expectations, EFA of the AHEAD items identified a 2-factor solution where “skills” and “values” formed separate factors. Internal consistency of AHEAD (overall and its 2 factors) was excellent and all items loaded significantly (factor loadings exceeded 0.40). Nevertheless, 4 items loaded differently from their designated factors (Table 2). Specifically, “values” items “courage”, “inspiration”, and “perseverance” loaded highly and specifically on the “skills” factor whilst “skills” item “interpersonal skills” loaded moderately on “values” factor.

How do we explain these discrepant results? Reviewing the literature, a few themes emerged. Our findings regarding item “courage” reveal that the circumstances in which courage is displayed in the workplace may have influenced how our participants have rated it. Indeed, business research has shown that courageous action involves calculated risk-taking and these decisions require careful deliberation and preparation [20]; hence, courage may be less of a pre-existing personal trait [21] but more of a skill that could be “learned, practiced, and grown alongside our responsibilities” [22].

As regards item “inspiration”, Bass [23] has emphasized that inspirational leadership focuses on communicating a compelling vision for the team, expressing confidence in team members, and energizing the team. Specifically, by communicating a vision, inspirational leaders can reinforce the common goals of the team; by expressing confidence in group members, they can enhance the group’s distinctiveness and prestige; and by energizing group members, they can encourage more interpersonal interaction among team members [24]. Thus, the ability to inspire depends not only on the leader’s charisma, but also the leader’s communication and implementation skills [23–25]. Also, given these skills are also more salient and measureable than one’s intrinsic character, it could be argued that inspiration may be skill-based.

Likewise, perseverance could be considered a skill because it depends on several factors that can be learnt. Research shows that grit, a function of perseverance and passion, is associated with educational attainment and age [26]. This association indicates that perseverance may increase with experience and hence, perseverance is not wholly intrinsic in an individual. Furthermore, the authors suggest that perseverance depends not only on motivation, commitment, or zeal, but it also depends on the capacity for hard work [26].

Our findings regarding “interpersonal skills” show that empathy wields a strong influence on interpersonal skills [27–29] and that the lack of interpersonal skills, or in other words, antisocial behavior – can be characterized by a lack of empathy and a possible inability to understand the emotions of others [29]. And yet, empathy is a significant factor associated with clinical competence and patient outcomes [30] and crucial for social functioning because interpreting such signals allows one to evaluate when and how to avoid social conflict [27]. In our study, the “interpersonal skills” item loaded moderately on both “skills” and “values” factors (Table 2), and this finding seems to agree with a qualitative study conducted on physicians which reported that empathy were considered both a personality characteristic and a skill [31].

### Convergent and discriminative validity

In our study, moderate correlations were demonstrated between AHEAD and LPI scores (Table 3). To the extent that the LPI is a well-established and validated measure of leadership competency [10], these correlation results suggest that AHEAD was assessing a similar underlying construct as the LPI, thereby providing evidence for its convergent validity. More importantly, AHEAD outperformed LPI in terms of discriminative validity (Fig. 1).

AHEAD scores may be more discriminative because given that the instrument was developed by AHPs, its conceptual focus may be more relevant to the AHP compared with the LPI. Furthermore, having to justify AHEAD ratings by citing examples may require participants to give a more accurate and honest view of themselves. In contrast, the LPI may be less favourable as a discriminative measure because the comparatively simpler way of rating oneself with LPI may influence respondents to rate themselves more hastily which may have introduced some discrepancy in results. This is especially so for junior AHPs, who may have rated themselves more highly than they would have if they had to produce supporting evidence. Finally, the AHEAD scores may be more discriminative because individual items descriptors were provided in the assessment form whereas only domain descriptors were available for the LPI. Conceivably, the detailed AHEAD item descriptors may bring respondents and their supervisors to a common level of understanding, which in turn, would have enhanced the predictive accuracy of the AHEAD scores.

### Limitations

Our study has limitations. First, our sample size was modest but this limitation may be countered by the few number of factors that we examined, the relatively large number of items per factor, and the moderately-high factor loadings [32]. Thus, based on the simulation results by Mundfrom et al. [32], we believe our sample size was at least adequate. Second, our focus on AHP leaders may limit study generalizability to this subset of AHPs. However, by limiting our study to AHPs who had the potential to lead and excel, we have provided a more robust test of convergent and discriminative validity of the AHEAD instrument. Third, we studied only AHPs so it remains uncertain if our findings are applicable to other healthcare professionals such as nurses, physicians, and hospital administrators. This limitation must, however, be balanced against the fact that the AHEAD instrument is one of the few leadership competency instruments developed in and for healthcare professionals. Accordingly, future studies should evaluate whether AHEAD could be used (or modified for use) in other healthcare disciplines or settings, preferably adopting a confirmatory factor analysis approach. Fourth, this cross-sectional study focused on convergent and discriminative validity of AHEAD; hence, it would be informative to evaluate, in a longitudinal fashion, the predictive validity of AHEAD scores with future important leadership outcomes – for example, leadership self-efficacy and follower outcomes and satisfaction [33].

## Conclusion

In conclusion, we have developed and validated the AHEAD instrument to evaluate leadership competency in existing and emerging AHP leaders. Although needing further validation, it is our hope that AHEAD will not only be used as an assessment tool, but it will also play a key role in an AHP’s leadership development.

## Additional files

Additional file 1:
**AHEAD Domains and Competencies. **(PDF 190 kb) Additional file 2:
**AHEAD Checklist and Resource Guides. **(XLS 3787 kb)

## Abbreviations

AHEAD: Aspiring leaders in Healthcare - Empowering individuals, Achieving excellence, Developing talents
AHP: Allied Health Professional
LPI: Leadership practices inventory
EFA: Exploratory factor analysis
c-index: Concordance index
