# Supplementary material for: Development and preliminary validation of a leadership competency instrument for existing and emerging allied health professional leaders
Source: BMC Health Serv Res. 2016 Feb 19;16:64. doi: 10.1186/s12913-016-1301-1 (PMC4759728; doi:10.1186/s12913-016-1301-1)
Supplement: Additional file 1: — AHEAD Domains and Competencies. (PDF 190 kb) [file 12913_2016_1301_MOESM1_ESM.pdf]

AHEAD Domains & Competencies

**VALUES**		Collegiality	Good leaders have the ability to work well and build trust with their staff as well as people that they come into contact with. They are approachable and make people feel comfortable with their sincerity and calm demeanor. They minimise conflict within the team and inspire warmth and affection.
		Commitment	Good leaders have the discipline to work towards the team's vision single-mindedly. They take ownership of every opportunity to meet expectations and requirements. They also anticipate the needs of those around them and offer assistance to the best of their ability.
		Compassionate	Good leaders understand another person's point of view by being sensitive and responsive to their needs. They are also empathetic to the feelings, values, interests and well-being of others and are able to put themselves into others' shoes.
		Courageous	Good leaders dare to take risks, try new things and stand up for what they believe in. They take responsibilities for their actions and embrace errors as a way to learn more. They guide others towards new visions, through tough times and over seemingly insurmountable hurdles.
		Humility	Good leaders with humility recognize that they are no better or worse than other members of the team. A humble leader is not self-effacing but rather tries to elevate everyone. They are never boastful of their achievements and competencies and continuously seek self-improvement.
		Impartiality	Impartiality means dealing with others consistently and justly. Good leaders treat each and every one of their staff fairly, without favoritism. They check all the facts and hear everyone out before passing judgment or jumping to conclusions based on incomplete evidence. They will use their authority in a wise and appropriate manner that befits their position rather than for the purpose of self-gratification. When people feel they that are being treated fairly, they reward a leader with loyalty and dedication.
		Inspirational	Good leaders bring out the best in their people by giving them a drive to achieve their dreams. They are able to motivate and draw people to them through persuasive communication of their visions and passions. "People want to be inspired...being inspiring is usually just a matter of communicating clearly and with passion." Being inspiring means telling people how your organization is going to change the world by showing them the big picture, helping them see beyond a narrow focus and understand how their

Correspondence: Ms. Ang Hui Gek, PPA(G), F.P.S.S., BSc (Pharm), Grad Dip Clin Pharm, MBA, Director, Allied Health Division, Singapore General Hospital, Singapore 169608 Tel.: +65 6326 6101; fax: +65 6220 2780. E-mail: ang.hui.gek@sgh.com.sg

			part fits into the big picture.
		Integrity	Integrity is the integration of outward actions and inner values. A person of integrity is the same on the outside and on the inside. Good leaders' moral values and actions are always in tandem with each other. They are able to discern between right and wrong and make decisions that are sound. They will not compromise principles, or even ideas, to please. Their integrity is what wins the trust of their people. Such an individual can be trusted because he or she never veers from inner values, even when it might be expeditious to do so.
		Perseverance	Good leaders are resilient and remain calm under stressful situations. They can direct their actions and those of the team towards achieving their goals despite facing numerous difficulties. They possess the discipline to keep to schedules and deadlines and display a high level of dedication to their work.
**SKILLS**	**Creative Innovator**	Change Management	As Peter Senge, an expert on managing organizations, says, "Everything is in motion, continually changing, forever adapting." Effective leaders recognize the value of change. Changes need to be anticipated and responded to if growth is going to continue. And that applies to minor changes, as well as major ones. Good leaders act as a change agents to identify opportunities for change, convince and gain commitment from the key stakeholders as well as ensure successful implementation. They champion change in the organization by introducing new ideas and concepts. At the same time, they overcome resistance to change by managing the expectations of all involved.
		Creative Thinking Skills	Creativity is the ability to think differently, to go outside of the box that constrains solutions. Creativity gives leaders the ability to see things that others have not seen and thus lead followers in new directions. Good leaders are able to make connections across seemingly unrelated questions, problems, or ideas by questioning. They have an ability to ask "what if", "why", and "why not" and constantly challenge the status quo to open up the bigger picture while experimenting with new concepts. They are also receptive to new ideas/suggestions from their staff.
	**Effective Decision Maker**	Decision Making Skills	Good leaders make practical and effective decisions in a timely fashion, even in situations of uncertainty and ambiguity. They ask themselves pertinent questions, list and evaluate their options by constructing best and worst scenarios and assess their chances of success and

			consequences of failure. Their decision making is served by openness and transparency whereby the reasons for making the decision are accessible and assessable. Leaders retain accountability towards their organization's stakeholders by taking responsibility for the outcomes that result from their choices.
	**Efficient Administrator**	Budgeting / Financial Planning	Financial planning is the continuous process of directing and allocating financial resources to meet the organization's strategic goals and objectives realistically. Regular review of budgets and cash flows answers three questions: "How are we doing?", "How much of the budget is left?" and "What will it look like at the end of the year?" Good leaders understands the need for clear and well justified workplans and budgets as it will enable the tracking of incomes and expenditures for the smooth and efficient running of projects. Regular monitoring of finances will allow one to spot trends and identify areas that can be improved or optimised.
		Organizational Awareness	Good leaders recognize the values, cultures and "power relationships" in the organization, and its effect on people's actions and behaviors. They are able to identify the real decision makers, political forces at work and know how to use this knowledge to their advantage in leading their team.
		Talent & Performance Management	Good leaders are focused on the performance of an individual by developing their staff's competencies and skillsets. They set performance expectations at work and provide constructive and consistent feedback to their staff as they understand what motivates different individuals to want to succeed. They are able to provide career development opportunities and guidance to enable their staff to reach their full potential. They identify staff with high potential and groom them for leadership positions, thus ensuring continuity of succession planning.
		Project Management	Good leaders plan, organize, secure and manage resources to bring about the successful completion of specific project goals and objectives within the constraints of time, cost, and performance requirements. They communicate effectively with their team, streamline processes, organize people through getting the right people to do the right job and manage risk to achieve quality outcomes while meeting objectives of all stakeholders involved.
	**Inspiring Coach**	Facilitating	Good leaders facilitate by setting goals, giving direction and understanding their team's working dynamics to bring the team's achievements to greater heights. They understand the individuality of team members and the dynamics of group interaction, thus creating a conducive

			social and psychological working environment.5 By knowing each team member well enough to be able to assess their strengths and weaknesses, they are able to use each team member to their fullest potential within the context of the team and make the team experience a positive one.
		Mentoring	Mentoring is a power free, two-way mutually beneficial learning situation where the mentor provides advice, teaches and, freely sharing their own experiences and skills with the mentees. Leaders as mentors help their mentees to acquire self-awareness and self-directiveness, develop a sense of purpose and experience success. Besides work-related issues, they are also personally involved as a friend who cares about mentee's long term development.
		Motivating	A positive attitude is a must in highly motivated leaders. Not only must leaders be positive, they must also be determined to change negative experiences of the group into growing experiences in order to lift the team's morale. Successful leaders are highly driven in their causes and can foster that same enthusiasm in their staff by providing an environment that encourages motivated members. They possess high energy levels, create task excitement and are catalysts for positive action. They understand their employees' needs, have the gift for verbal persuasion to get their staff to "buy in" to their ideas and inspire them to greater heights.
	**Strong Communicator**	Interpersonal Skills	Good leaders have the ability to relate and work well with people that they come into contact with. They possess a high level of Emotional Intelligence (EI) which is a combination of self-management and social skills that can transform and optimize individual or team performance. Leaders "have the emotional intelligence or EI traits to control his emotions. Often, ordinary persons break under pressure or give up when pushed a little out of their comfort zone. On the contrary, a leader, in such a situation, takes up the task as a challenge and an opportunity to learn. The truest and the best leadership qualities are always witnessed at demanding times."
		Inquiry	Leaders "...have the ability to listen more and talk less. It is more important to listen to the issues that are being raised instead of expressing your opinion about them. Some individuals have the misconception that a good leader talks as much as possible." "The purpose of listening is to connect and learn." Effective leaders realize that listening provides them with a deeper understanding of the needs of those that surround them and listen not only for words, but

			also feelings, meanings and undercurrents. It also gives them a greater insight into the issues that must be addressed." Good leaders are good listeners. They draw upon the expertise and ideas of people around them to improve their decision making as leaders who listen well make better decisions.
		Presentation Skills	Good leaders always speak in the listeners' language, which is what helps them to communicate better. An efficient leader is never stuck up in the jungle of jargon, which leaves his audience baffled. He is always clear and concise, while communicating his motives and plans. They are able to present ideas with professionalism, in a persuasive manner. "Preparation and knowledge are the pre-requisites for a successful presentation..." Leaders prepare for presentations by setting the objectives and desired outcomes, identifying target audience, preempting possible questions and objections, and practice, practice, practice.
		Writing Skills	Good leaders are able to communicate their ideas through writing in a clear and persuasive manner. The writing process helps leaders to better understand their decision making methods and provides deeper insights into making good decisions. Writing allows leaders to organize and clarify their thoughts and help them to privately develop their perspective of issues in a way that can be communicated to others. By putting their thoughts into words, they give people a concrete standard that they can reference and align everyone’s efforts toward the same goal. It is also much easier to hold people accountable for something that you’ve communicated in writing.
	**Strategic Visionary**	Foresight / Strategy	Murray & Mann stated that a proficient leader "has a vision, an intense focus on outcome and results, a realistic strategy to carry out the vision and the ability to communicate the vision and rally support of others" (Williams, 1993, p. 87). As such, leaders need to be intimately involved with setting the strategic direction based on a thorough assessment of the organization and its external environment. Good leaders select the right strategy for the right situation, even when the pressure is overwhelming. They are well-organized and recognize the importance of setting aside time to plan and think about the future. This enables them to think two steps ahead and be rarely caught off guard. Besides having a clear vision, leaders must be willing to share their vision with others as people need to know that a leader has a strong vision for the future and a strong plan for going forward. It is

			important that leaders communicate their vision in a clear and passionate manner in order to encourage buy-in.
	**Expert Practitioner**	Domain Knowledge	Competent leaders must "possess the skills and abilities required to do the work or have the potential to learn whatever is necessary." Good leaders have the necessary tools required for them to perform their roles well and understand when and how to use these tools appropriately and effectively. In this way, they are able to "deliver the right outcome at just the right time". In order for leaders to gain support from their subordinates, leaders have to recognize as "competent". This does not mean that leaders have to be the foremost experts in every area of their organization, rather, they will have to showcase a certain level of achievements in their own area(s) of practice.
